# Cerebral malaria: A life-threatening complication

**DOI:** 10.1590/0037-8682-0186-2021

**Published:** 2021-04-28

**Authors:** Tumay Bekci, Ismet Mirac Cakir, Serdar Aslan

**Affiliations:** 1 Giresun University, Faculty of Medicine, Department of Radiology, Giresun, Turkey.

**FIGURE 1:**
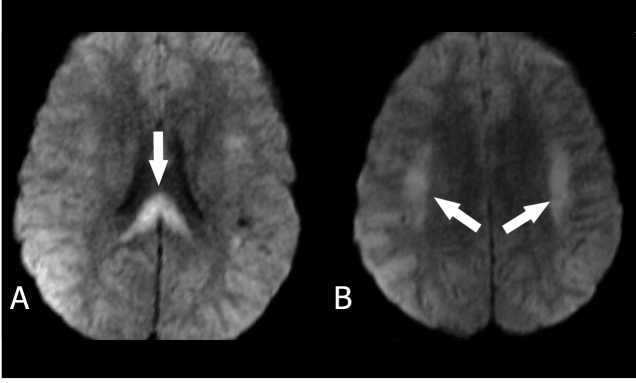
Diffusion-weighted magnetic resonance imaging demonstrated restricted diffusion on **(A)** splenium of the corpus callosum and **(B)** bilateral subcortical areas (arrows).

A 25-year-old man was admitted to our hospital with complaints of hematuria, abdominal pain, vomiting, and high fever. Upon admission, patient’s fever was 38.6 °C, platelet count was 22000 × 10^9^ /L, hemoglobin level was 100 g/L, and retinal hemorrhages were observed on ophthalmoscopic examination. The patient’s history revealed that he had traveled to Chad six days prior. With the initial diagnosis of malaria, a blood smear was performed to identify the malaria parasites. The patient was started on medical treatment. However, on the fourth day of admission, the patient showed neurological signs and confusion. With suspicion of cerebral involvement, brain diffusion-weighted magnetic resonance imaging (DW-MRI) was performed. DW-MRI (Figure 1) demonstrated restricted diffusion in the bilateral subcortical areas and splenium of the corpus callosum. The patient was treated with antiepileptic, antimalarial, and antiaggregant and anticoagulant drugs. On the eleventh day of admission, the patient was discharged with full recovery.

Cerebral malaria is a life-threatening complication of *Plasmodium falciparum* infection. The clinical hallmark of cerebral malaria is impaired consciousness, with coma being the most severe manifestation. Hemorrhages are thought to occur when sequestered *Plasmodium*-infected erythrocytes occlude the cerebral capillaries and small veins. Hence, this pathological process may lead to infarction^1^. Without treatment, cerebral malaria is invariably fatal. DW-MRI sequences are extremely sensitive for detecting cytotoxic edema and have been widely used in the assessment of cases with cerebral manifestations^2^. Therefore, radiologists and clinicians should be familiar with the imaging findings of cerebral malaria.
